# A new non-invasive index for prognosis evaluation in patients with aortic stenosis

**DOI:** 10.1038/s41598-020-63777-z

**Published:** 2020-04-30

**Authors:** Hui Wen Sim, Nicholas Jinghao Ngiam, Liang Zhong, Benjamin Yong-Qiang Tan, Lyndon Y Low, Andie Hartanto Djohan, Elaine Boey, William Kok Fai Kong, Ru San Tan, Kian Keong Poh

**Affiliations:** 1 National University Heart Centre Singapore, 1E Kent Ridge Road, NUHS Tower Block, Level 9 Singapore, 119228 Singapore; 2 National Heart Centre Singapore, 5 Hospital Dr, Singapore, 169609 Singapore; 3 Duke-NUS Medical School, National University of Singapore, 8 College Road, Singapore, 169857 Singapore; 4 Department of Medicine, Yong Loo Lin School of Medicine, National University of Singapore, 1E Kent Ridge Road, NUHS Tower Block, Level 11, Singapore, 119228 Singapore

**Keywords:** Cardiology, Cardiovascular biology

## Abstract

The global left ventricular (LV) contractility index, dσ*/dt_*max*_ measures the maximal rate of change in pressure-normalized LV wall stress. We aim to describe the trend of dσ*/dt_*max*_ in differing severity of aortic stenosis (AS) with preserved left ventricular ejection fraction (LVEF) and the association of dσ*/dt_*max*_ with clinical outcomes in moderate AS and severe AS. We retrospectively studied a total of 1738 patients with AS (550 mild AS, 738 moderate AS, 450 severe AS) and preserved LVEF ≥ 50% diagnosed from 1^st^ January 2001 to 31^st^ December 2015. dσ*/dt_*max*_ worsened with increasing severity of AS despite preserved LVEF (mild AS: 3.69 ± 1.28 s^−1^, moderate AS: 3.17 ± 1.09 s^−1^, severe AS: 2.58 ± 0.83 s^−1^, *p* < 0.001). Low dσ*/dt_*max*_ < 2.8 s^−1^ was independently associated with a higher composite outcome of aortic valve replacement, congestive cardiac failure admissions and all-cause mortality (adjusted hazard ratio 1.48, 95% CI: 1.25–1.77, *p* < 0.001). In conclusion, dσ*/dt_*max*_ declined with worsening AS despite preserved LVEF. Low dσ*/dt_*max*_ < 2.8 s^−1^ was independently associated with adverse clinical outcomes in moderate AS and severe AS with preserved LVEF.

## Introduction

Aortic stenosis (AS) is the most common native valvular heart disease amongst the elderly population^1,2^. Aortic valve replacement (AVR) is a class I indication for symptomatic severe AS or asymptomatic severe AS with reduced left ventricular ejection fraction (LVEF) < 50%^3^. Recent studies have demonstratedthe benefit of early surgical interventionin AS when LVEF remains preserved^4–8^. However, measurement of LVEF is dependent on alteration in left ventricle (LV) loading condition and may not necessarily reflect the intrinsic contractile state of the LV^9–11^. A novel echocardiographic-derived global LV contractility index, dσ*/dt_*max*_ represents an integrated assessment of LV contractility, as it is primarily dependent on the outflow rate and wall volume of the LV chamber. dσ*/dt_*max*_ is a validated index that measures the maximal rate of change in pressure-normalized LV wall stress^12–16^. As compared to normal controls, dσ*/dt_*max*_ was found to be lower in heart failure patients with preserved LVEF^14,15^. To date, dσ*/dt_*max*_ has not been studied in pressure overload states. We aim to describe the trend of dσ*/dt_*max*_ in different severity categories of AS with preserved LVEF, and the association of dσ*/dt_*max*_ with clinical outcomes in moderate AS and severe AS with preserved LVEF.

## Methods

Consecutive patients with isolated AS and preserved LVEF ≥ 50% diagnosed on index echocardiography from 1^st ^January 2001 to 31^st ^December 2015 from a single-centre were retrospectively examined. Patients with other significant valvular lesions (of at least moderate severity) or any prior valvular interventions were excluded. They were divided into differing severity categories of AS based on the aortic valve area calculated by continuity equation: mild AS (aortic valve area > 1.5cm^2^), moderate AS (aortic valve area 1.0–1.5 cm^2^) and severe AS (aortic valve area < 1.0 cm^2^). Baseline demographics and echocardiographic parameters were documented. Global LV contractility index, dσ*/dt_*max*_ were calculated for all patients using non-invasive echocardiographic measurements. In patients with moderate AS and severe AS, clinical outcomes were obtained by review of medical records and outpatient attendance extracted up to 30^th^ November 2018 for analysis. Composite events were defined as a combination of AVR, congestive cardiac failure (CCF) admissions and all-cause mortality. The association of global LV contractility index, dσ*/dt_*max*_ with the composite outcome and its individual components were studied. Receiver operating characteristic curves were used to identify the optimal cut off value for dσ*/dt_*max*_ for composite clinical events. Ethics approval was obtained from the National Healthcare Group Domain Specific Review Board (DSRB) prior to the conduct of the study. This study complied with all DSRB requirements based on Declaration of Helsinki and the ethical principles in the Belmont Report. The DSRB requirements were also compliant with the guidelines stipulated by the Bioethics Advisory Committee. No patient identifiers were obtained during the study, and no tissue samples were collected. A waiver for the need for patient consent was obtained from DSRB.

Transthoracic echocardiography was performed by cardiac sonographers with standard echocardiography imaging system and analysed by qualified cardiologists. Blood pressures and heart rates were obtained at the start of the studies. Chamber dimensions, LV wall thickness, chamber volume, LV mass and biplane modified Simpson’s LVEF were measured using standard recommendations for LV chamber quantifications^17^. We classify severity of AS based the European Association of Cardiovascular Imaging and the American Society of Echocardiography recommendations on echocardiographic assessment of aortic valve stenosis^18^. Aortic valve area was calculated using continuity equation. The transaortic peak velocity and transaortic mean pressure gradient were obtained using continuous wave Doppler from multiple windows to obtain the maximum velocity. Left ventricular outflow tract (LVOT) diameter was measured on parasternal long axis view. LVOT peak velocity and velocity time integral were performed using pulse wave Doppler from apical 3-chamber view, 5 mm away from the aortic valve in the LVOT^18^. Assessment of diastolic function to derive mitral E velocity, mitral E/A ratio, mitral E wave deceleration time and septal E/e’ ratio were done based on current recommendations for LV diastolic function^19^. Septale’ velocity was obtained using pulse wave tissue doppler imaging at apical four chamber view with the sample volume at the basal septal region. During LV contraction, myocardial sarcomere activation generates myocardial wall stress that induces intracavity pressure during LV contraction, which can be measured by maximal *dP/dt*. Myocardial wall stress occurred prior to the rise of LV intracavitary pressure and can be potentially used as a measurement of ventricular performance. By using biomechanical model, LV wall stress is expressed as: *σ* = *P*(3*V*/2*V*_*m*_ + 1/2), where *σ* is wall stress, *P* and *V* are LV intracavitary pressure and volume, *V*_*m*_ is myocardial volume. Rearranging the equation, we get: σ* = *σ*/*P* = (3*V*/2*V*_*m*_ + 1/2). Analogous to *dP/dt*_*max*_, we defined dσ*/dt_*max*_ as:  dσ*/*dt*_*max*_ = 3(*dV*/*dt*)_*max*_/2*V*_*m*_, where σ* is *σ/P* or pressure-normalized wall stress, and *dV/dt*_*max*_ is the maximal flow rate. The latter is calculated from standard echocardiography (maximal LVOT velocity on pulse wave Doppler echocardiogram *V*_*peak*_ and LVOT area): *dV*/*dt*_*max*_ = *V*_*peak*_ x πD^2^/4, where D is the LVOT diameter measured in the 2-dimensional parasternal long axis. Myocardial volume (*V*_*m*_) is calculated from the quotient of LV mass (obtained from standard M-mode echocardiography) and myocardial density (assumed to be 1.05 g/ml)^14–16^.

### Statistical methods

Categorical data were expressed as frequency and percentages. Continuous variables were summarized as mean (±standard deviation). Chi-squared tests, Student’s t-tests and one-way analysis of variance (ANOVA) with post-hoc Bonferroni analyses were used to compare variables between groups. A receiver operating characteristic curve was constructed for dσ*/dt_*max*_ in predicting composite clinical outcomes (AVR, admissions for CCF and all-cause mortality) on subsequent follow-up. Youden’s index (J) was tabulated to determine the optimised cut off for this outcome. dσ*/dt_*max*_ was defined as low when it was below the cut-off value identified by the receiver operating characteristic analysis. Clinical outcomes were compared by the construction of Kaplan–Meier curves. To adjust for differences in baseline characteristics between groups, a multivariable Cox proportional hazards regression model was constructed to obtain the risk-adjusted association between dσ*/dt_*max*_ and composite clinical outcomes. Covariates that potentially may have had confounding effect on contractility index in predicting poor clinical outcomes [age, hypertension, diabetes, ischemic heart disease and echocardiographic markers of AS severity (such as aortic valve area and transaortic peak velocity)] were incorporated into the model. A two-tailed value of *p* ≤ 0.05 was used to reject the null hypothesis. Statistical analysis was done using SPSS 21.0 (SPSS Inc., Chicago IL, USA).

## Results

A total of 1738 patients with isolated AS [mild AS 550 (31.6%), moderate AS 738 (42.5%), severe AS 450 (25.9%)] were included in this study. Table 1 shows the baseline demographics and echocardiographic parameters of mild AS, moderate AS and severe AS. Age and gender were similar across all three categories of AS severity. Patients with severe AS had the lowest prevalence of hypertension, diabetes, ischemic heart disease and chronic kidney disease as compared to mild AS or moderate AS. The prevalence of hyperlipidemia, stroke/transient ischemic attack, atrial fibrillation, peripheral vascular disease and chronic obstructive pulmonary disease did not differ among the three groups. Severe AS had the highest transaortic peak velocity, highest transaortic mean pressure gradient, highest LV mass index, smallest LVOT diameter, lowest LVOT peak velocity, highest mitral E velocity and highest septal E/e’ ratio. There was a decline in the global LV contractility index, dσ*/dt_*max*_ with increasing severity of AS (mild AS: 3.69 ± 1.28 s^−1^, moderate AS: 3.17 ± 1.09 s^−1^, severe AS: 2.58 ± 0.83 s^−1^, *p* < 0.001). dσ*/dt_*max*_ was significantly different between the different categories of AS severity on post-hoc pairwise Bonferroni analysis.

**Table 1:** Baseline demographics and echocardiographic parameters of study population divided according to AS severity.

Variables: mean ± SD or n (%)	All N = 1738	Mild AS N = 550	Moderate AS N = 738	Severe AS N = 450	*p*-value
**Baseline demographics**					
Age (years)	72.4 (±12.6)	72.0 (±12.4)	73.0 (±12.5)	71.8 (±13.1)	0.183
Male	751 (43.2%)	295 (53.6%)	299 (40.5%)	157 (34.8%)	0.173
Hypertension	1276 (73.4%)	418 (76.0%)	563 (76.3%)	295 (65.6%)	<0.001
Hyperlipidemia	955 (54.9%)	305 (55.5%)	424 (57.5%)	226 (50.3%)	0.055
Diabetes	696 (40.0%)	239 (43.5%)	300 (40.7%)	157 (34.9%)	0.021
Stroke/TIA	313 (18.0%)	101 (18.4%)	144 (19.5%)	68 (15.1%)	0.154
Atrial fibrillation	219 (12.4%)	67 (12.2%)	95 (12.9%)	54 (12.0%)	0.887
IHD	590 (33.9%)	196 (35.6%)	267 (36.2%)	127 (28.2%)	0.012
PVD	79 (4.5%)	23 (4.2%)	33 (4.5%)	23 (5.1%)	0.776
CKD	443 (25.5%)	120 (21.8%)	141 (19.1%)	62 (13.8%)	0.004
COPD	69 (4.0%)	26 (4.7%)	30 (4.1%)	13 (2.9%)	0.329
Systolic BP (mmHg)	141 (±24)	142 (±23)	142 (±25)	138 (±24)	0.002
Diastolic BP (mmHg)	72.3 (±12.4)	73.1 (±12.8)	72.3 (±12.3)	71.4 (±12.0)	0.080
Heart rate (beats/min)	71.5 (±23.3)	71.4 (±15.8)	70.7 (±14.6)	71.5 (±15.9)	0.604
**Echocardiographic parameters**					
Aortic valve area (cm^2^)	1.3 (±0.5)	1.8 (±0.3)	1.3 (±0.1)	0.8 (±0.2)	<0.001
Transaortic peak velocity (cm/s)	284 (±84)	235 (±42)	264 (±53)	375 (±93)	<0.001
Transaortic mean pressure gradient (mmHg)	20.5 (±18.3)	13.1 (±12.8)	16.8 (±15.9)	35.5 (±19.2)	<0.001
LV mass index (g/m^2^)	109 (±32)	107 (±29)	105 (±30)	118 (±38)	<0.001
LVOT diameter (mm)	20.3 (±1.9)	21.1 (±1.8)	20.0 (±1.7)	19.8 (±1.9)	<0.001
LVOT peak velocity (cm/s)	104 (±22.6)	114 (±24)	103 (±21)	94 (±18)	<0.001
LV end diastolic volume index (ml/m^2^)	60.8 (±17.6)	60.4 (±16.8)	60.6 (±17.3)	61.8 (±19.1)	0.379
Stroke volume index (ml/m^2^)	40.9 (±11.6)	41.0 (±11.4)	40.8 (±11.4)	40.8 (±12.2)	0.936
Mitral E velocity (cm/s)	86.8 (±31.6)	83.5 (±28.8)	87.2 (±31.8)	89.7 (±34.3)	0.007
Mitral E/A ratio	0.7 (±8.3)	0.6 (±5.5)	0.7 (±10.1)	1.1 (±7.9)	0.646
Mitral E wave deceleration time (ms)	220 (±85)	222 (±73)	220 (±73)	217 (±80)	0.574
Septal E/e’ ratio	15.6 (±8.5)	14.0 (±6.6)	16.0 (±8.8)	16.8 (±10.2)	<0.001
dσ*/dt_*max*_ (s^−1^)	3.18 (±1.17)	3.69 (±1.28)	3.17 (±1.09)	2.58 (±0.83)	<0.001

AS: aortic stenosis; BP: blood pressure; CKD: chronic kidney disease; COPD: chronic obstructive pulmonary disease; IHD: ischemic heart disease; LV: left ventricle; LVOT: left ventricular outflow tract; PVD: peripheral vascular disease; SD: standard deviation; TIA: transient ischemic attack; dσ*/dt_*max*_: global left ventricular contractility index.

During a median follow up of 3.58 years (interquartile range: 1.11–6.06 years) in patients with moderate AS and severe AS (n = 1188), composite outcomes occurred in 625 (52.6%) patients [AVR 201 (16.9%), CCF admissions 119 (10.0%), all-cause mortality 439 (37.0%)]. The cumulative mortality rates for moderate AS and severe AS were 34.6% and 40.9%, respectively. By using receiver operating characteristic analysis, we identified an optimized cut-off of < 2.8 s^−1^ for dσ*/dt_*max*_ to be associated with occurrence of composite outcome with an area under the curve of 0.61, 95% CI: 0.58–0.64, *p* < 0.001, sensitivity of 59% and specificity of 59%. A total of 601 (50.6%) patients had dσ*/dt_*max*_ < 2.8 s^−1^ and 587 (49.4%) had dσ*/dt_*max*_ ≥ 2.8 s^−1^. Patients with low contractility index (dσ*/dt_*max*_ < 2.8 s^−1^) were similar in terms of age and clinical profile, except for a higher incidence of chronic kidney disease (21.6% vs 13.1%) and ischemic heart disease (36.6% vs 29.6%). The LVEF was similar in spite of a lower contractility index (59.9 ± 4.4% vs 63.0 ± 2.2%, *p* = 0.337). Patients with lower contractility index (< 2.8 s^−1^) also had smaller aortic valve area, higher transaortic mean pressure gradients and peak velocity and lower LVOT velocity. The LV end diastolic volume index and stroke volume index were both significantly higher in patients with lower contractility index (Table 2).

**Table 2:** Baseline demographics and echocardiographic parameters of study population divided according to contractility index.

Variables: mean ± SD or n (%)	All N = 1188	Low contractility index (<2.8 s^−1^) N = 601	High contractility Index (≥2.8 s^−1^) N = 587	*p*-value
**Baseline demographics**				
Age (years)	72.5 (±12.7)	72.2 (±12.2)	72.9 (±13.2)	0.330
Male	473 (39.8%)	266 (44.3%)	207 (35.3%)	0.173
Hypertension	858 (72.2%)	438 (72.9%)	420 (71.6%)	0.525
Hyperlipidemia	650 (54.7%)	324 (54.0%)	326 (55.5%)	0.595
Diabetes	457 (38.5%)	240 (39.9%)	217 (37.0%)	0.293
Stroke/TIA	212 (17.8%)	119 (19.8%)	93 (15.8%)	0.075
Atrial fibrillation	167 (14.1%)	81 (13.5%)	68 (11.6%)	0.325
IHD	394 (33.2%)	220 (36.6%)	174 (29.6%)	0.011
PVD	246 (20.7%)	33 (5.5%)	213 (3.9%)	0.199
CKD	203 (17.1%)	126 (21.6%)	77 (13.1%)	<0.001
COPD	43 (3.6%)	22 (3.7%)	21 (3.6%)	0.934
Systolic BP (mmHg)	140.6 (±24.5)	141.5 (±25.8)	139.6 (±23.1)	0.176
Diastolic BP (mmHg)	72.0 (±12.2)	72.4 (±12.7)	71.5 (±11.6)	0.212
Heart rate (beats/min)	71.0 (±15.1)	70.1 (±15.7)	71.9 (±14.5)	0.045
**Echocardiographic parameters**				
Aortic valve area (cm^2^)	1.1 (±0.3)	1.0 (±0.3)	1.2 (±0.2)	<0.001
Transaortic peak velocity (cm/s)	306.0 (±89.0)	318.7 (±102.0)	292.9 (±71.1)	<0.001
Transaortic mean pressure gradient (mmHg)	23.9 (±19.5)	26.7 (±23.9)	21.0 (±12.9)	<0.001
LV mass index (g/m^2^)	109.6 (±33.4)	129.2 (±32.4)	90.0 (±20.4)	<0.001
LV Ejection Fraction (%)	61.5 (±3.3)	59.9 (±4.4)	63.0 (±2.2)	0.337
LVOT diameter (mm)	19.9 (±1.8)	19.8 (±1.8)	19.9 (±1.7)	0.110
LVOT peak velocity (cm/s)	99.6 (±20.4)	94.2 (±18.9)	105.1 (±20.4)	<0.001
LV end diastolic volume index (ml/m^2^)	61.0 (±18.0)	66.5 (±18.8)	55.5 (±15.3)	<0.001
Stroke volume index (ml/m^2^)	40.8 (±11.7)	43.6 (±12.1)	38.0 (±10.6)	<0.001
Mitral E/A ratio	0.7 (±0.3)	0.7 (±0.3)	0.7 (±0.3)	0.769
Mitral E wave deceleration time (ms)	218.4 (±75.7)	219.0 (±74.8)	217.7 (±76.7)	0.779
Septal E/e’ ratio	16.0 (±9.2)	16.9 (±10.6)	15.2 (±7.7)	0.003
dσ*/dt_*max*_ (s^−1^)	2.95 (±1.04)	2.19 (±0.41)	3.72 (±0.91)	<0.001

AS: aortic stenosis; BP: blood pressure; CKD: chronic kidney disease; COPD: chronic obstructive pulmonary disease; IHD: ischemic heart disease; LV: left ventricle; LVOT: left ventricular outflow tract; PVD: peripheral vascular disease; SD: standard deviation; TIA: transient ischemic attack; dσ*/dt_*max*_: global left ventricular contractility index.

**Figure 1:**
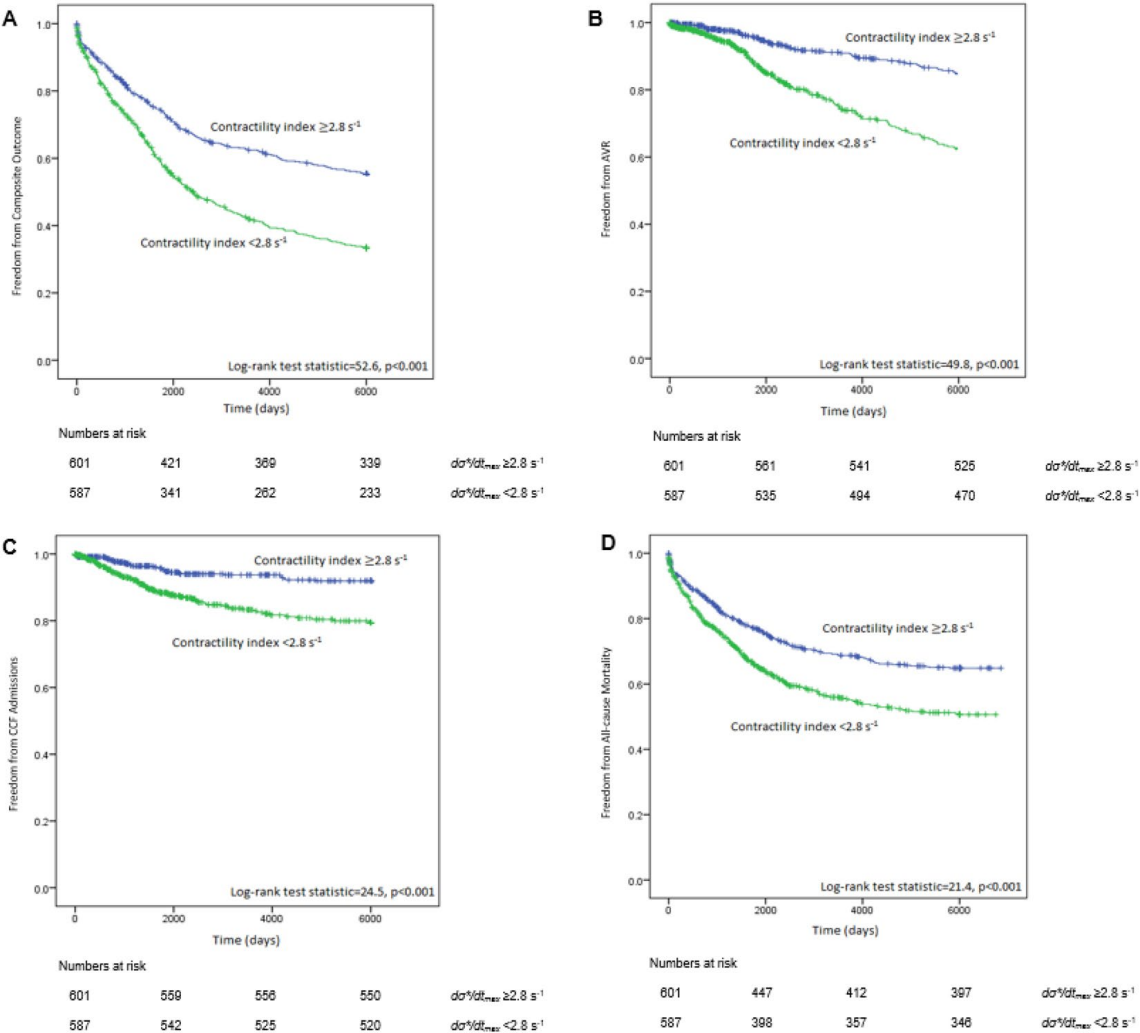
Kaplan-Meier event-free survival curve according to dσ*/dt_*max*_ cut off value of < 2.8 s^−1^ vs ≥ 2.8 s^−1^. (**A**) Composite outcome of AVR, CCF admissions and all-cause mortality, (**B**) AVR, (**C**) CCF admissions, (**D**) All-cause mortality. dσ*/dt_*max*_: global left ventricular contractility index; AVR: aortic valve replacement; CCF: congestive cardiac failure.

Composite outcomes occurred in 371 (61.7%) patients with dσ*/dt_*max*_ < 2.8 s^−1^[AVR 133 (22.1%), CCF admissions 80 (13.3%), all-cause mortality 251 (41.8%)] as compared to 254 (43.3%) patients with dσ*/dt_*max*_ ≥ 2.8 s^−1^ [AVR 68 (11.6%), CCF admissions 39 (6.6%), all-cause mortality 188 (32.0%), *p* < *0.001*]. On Kaplan-Meier survival time-to-event analysis, patients with dσ*/dt_*max*_ < 2.8 s^−1^ had significantly higher occurrence of composite events (log rank test 52.6, *p* < 0.001) and its individual components of AVR (log rank test 49.8, *p* < 0.001), CCF admissions (log rank test 24.5, *p* < 0.001) and all-cause mortality (log rank test 21.4, *p* < 0.001) as compared to dσ*/dt_*max*_ ≥ 2.8 s^−1^. (Fig. 1) On multivariable Cox regression, dσ*/dt_*max*_ < 2.8 s^−1^ remained independently associated with the occurrence of composite outcomes (adjusted HR 1.48, 95% CI: 1.25–1.77, *p* < 0.001) (Table 3).

**Table 3:** Multivariable Cox regression analysis showing dσ*/*dt*_*max*_ independently associated poor composite clinical outcomes in patients with moderate aortic stenosis and severe aortic stenosis.

Variables	Hazard Ratio (95% CI)	*p*-value
Age (years)	1.02 (1.02–1.03)	<0.001
Hypertension	1.04 (0.86–1.25)	0.163
Diabetes mellitus	1.19 (1.00–1.40)	0.051
Ischemic heart disease	1.34 (1.13–1.58)	0.001
Aortic valve area (cm^2^)	0.53 (0.35–0.81)	0.003
Transaortic peak velocity (cm/s)	1.002 (1.001–1.004)	<0.001
dσ*/dt_*max*_ < 2.8 s^−1^	1.48 (1.25–1.77)	<0.001

AVR: aortic valve replacement; CCF: congestive cardiac failure; CI: confidence interval; dσ*/dt_*max*_: global left ventricular contractility index.

## Discussion

The main findings of this study were as follows: (i) There was a declining trend of the global LV contractility index, dσ*/dt_*max*_ with worsening severity of AS (from mild AS to severe AS) despite having preserved LVEF ≥ 50%. (ii) Low dσ*/dt_*max*_ < 2.8 s^−1^ was associated with higher composite clinical outcomes of AVR, CCF admissions and all-cause mortality. (iii) After adjusting for age, cardiovascular risk factors, and echocardiographic markers of AS severity (such as aortic valve area and transaortic peak velocity), low contractility index (< 2.8 s^−1^) remained independently associated with poor clinical outcomes on multivariable Cox regression.

The global LV contractility index, dσ*/dt_*max*_ was conceived de novo using basic mechanical engineering principles and geometrical assumptions of the LV. It estimated the maximum rate of development of LV wall stress in relation to LV pressure (LV wall stress was normalized to LV pressure in the formula to obviate the need for invasive LV pressure measurement). As LV wall stress is generated intrinsically by sarcomere contraction that in turn transmits to extrinsic LV pressure, the index is based on the hypothesis that the capacity of the LV to develop intramyocardial stress to eject blood volume constitutes LV contractility^12^. dσ*/dt_*max*_ is dependent on the maximal outflow rate from the ventricle at ejection phase, normalized to myocardial volume^20,21^. To date, there is no technique to measure LV wall stress directly. dσ*/dt_*max*_ takes into account the combination of LV wall stress (wall thickness, geometry, chamber pressure, sarcomere contraction) and wall material properties. In experimental studies, it was found to be insensitive to preload and afterload changes within physiological limits and may potentially reflect intrinsic LV contractility^12,13^. Conveniently, we were able to calculate dσ*/dt_*max*_ non-invasively using routine echocardiographic measurements (by using LVOT diameter, LVOT peak velocity and myocardial volume) without the need for specialized acquisition, software or intensive analysis. *Zhong L. et al*. studied dσ*/dt_*max*_ in heart failure patients and found that heart failure patients with preserved LVEF had lower dσ*/dt_*max*_ at 2.57 s^−1^ as compared to 4.30 s^−1^ in normal controls^14^.

Proper assessment of LV function in AS is essential in clinical decision making. The current guideline recommends AVR for symptomatic patients or asymptomatic patients when LVEF is ≤ 50%^3^. Due to its load-dependent nature, LVEF may not be a sensitive marker of global LV systolic function^22^. In chronic AS, LV hypertrophy and increased LV mass developed as adaptive responses to normalize increased wall stress (from pressure overload)^23^. These LV geometric changes allow for preservation of LVEF despite depressed myocardial shortening^22,24^. As the disease progresses, LV remodelling leads to sub-endocardial ischemia and myocardial fibrosis that reduces global LV systolic function^8,25–28^. Therefore, we need to detect subtle impairment in LV performance before the deterioration of LVEF. For better quantification of LV systolic function, studies had looked at newer indices such as LV systolic longitudinal function, mitral annulus systolic displacement, myocardial systolic velocity s from tissue Doppler imaging and myocardial deformation parameters (strain, strain rate or twist measurement)^29–33^. Longitudinal strain had been commonly used to evaluate LV mechanics in AS and found to be abnormal in AS patients despite preserved LVEF^34–36^. Our data were accrued over a long period and most images are no longer amendable to global longitudinal strain (GLS) analysis. Indeed, the measurement of GLS has been demonstrated to be useful in assessing subclinical LV dysfunction, and subsequent decline in the preserved LVEF. However, measurement of GLS may be time-consuming, and requires either off-line analysis or specialised software systems that may only be installed on machines with higher specifications. It is operator dependent with high inter-observer variability. These factors may limit its use in certain centres. By contrast, the novel contractility index we studied was based on physiological principles and can be easily derived from readily available echocardiographic parameters. In our study, dσ*/dt_*max*_ was lowest in severe AS at 2.58 s^−1^, as compared to 3.17 s^−1^ in moderate AS and 3.69 s^−1^ in mild AS, indicating the presence of sub-clinical myocardial dysfunction with increasing pressure overload leading to LV remodelling. Severe AS has the lowest dσ*/dt_*max*_ despite having a lower prevalence of hypertension and ischemic heart disease as compared with mild AS and moderate AS.

Risk stratification for proper timing of AVR in AS is complex and challenging^4–6^. Traditionally, symptom status, aortic valve area, aortic jet velocity, transaortic mean pressure gradient were important predictors of adverse outcome in AS. More recently, *Cioffi G. et al*. showed that asymptomatic severe AS with inappropriate LV hypertrophy had a 4.5-fold higher risk of death, AVR or hospital admissions; while *Lancelloti P. et. al*. found an integrated measurement of peak aortic jet ≥ 4.4 m/s, LV longitudinal myocardial impedance ≤ 15.9%, valvuloarterial impedence ≥ 4.9 mmHg/ml/m^2^ and indexed left atrial area ≥ 12.2cm^2^/m^2^ improved risk stratification in AS^37,38^. Non-echocardiographic tools such as computed tomographic aortic valve calcium scoring and magnetic resonance imaging focal mid-wall fibrosis were predictive of mortality in AS^39,40^. Our study found that reduced global LV contractility index, dσ*/dt_*max*_ < 2.8 s^−1^ in patients with moderate and severe AS was associated with higher risk of AVR, CCF admissions and death despite preserved LVEF. These observations were likely due to the presence of myocardial fibrosis and poorer contractile reserve in patients with lower baseline dσ*/dt_*max*_ that accounted for the worse clinical outcomes. Current evidence demonstrated that the assessment of LV function solely by LVEF measurement is not adequate. Hence, dσ*/dt_*max*_ may be useful as an additional tool in risk stratification of AS and help to pre-select patients who may benefit from early intervention.

Transcatheter aortic valve replacement (TAVR) has been shown to reduce mortality and morbidity and evolved as a treatment option for patients at intermediate surgical risk^41^. There has been a growing interest in risk stratification and outcome prediction strategies for TAVR candidates beyond the Society of Thoracic Surgeon’s risk score and EUROscore^42^. By the time these patients develop LV dysfunction, they may have poorer outcome post AVR^8,25–28^. Our study cohort spanned 15 years (2001 to 2015) and our TAVR program was started in 2010. Out of 1188 patients with moderate and severe AS at baseline, 171 subsequently underwent surgical AVR while only 30 (2.5% of the total cohort) underwent TAVR. While we have observed with this cohort that dσ*/dt_*max*_ was sensitive for early LV dysfunction and carried prognostic implications, we had not used it to plan for TAVR. It will require a prospective strategy trial to validate its utility in this setting.

Our study observed high mortality rates that commensurate with prior large registry of AS. In a recently published paper by Strange *et al*., AS was characterized in 122,809 male patients (mean age 61 ± 17 years) and 118,494 female patients (mean age 62 ± 19 years), and 16,129 (6.7%), 3,315 (1.4%), and 6,383 (2.6%) patients had mild, moderate, and severe AS, respectively^43^. 5-year mortality rates for patients with mild AS, moderate AS and severe AS were 43%, 56%, and 67%, respectively. 1-year mortality rates for patients with mild AS, moderate AS and severe AS were 15%, 21%, and 29%, respectively; implying death events were front-loaded. Our population is older, and cumulative mortality rates at median 3.6 years follow-up for mild AS, moderate AS and severe AS were 29.3%, 34.6% and 40.9%, respectively. Specifically, the mortality rate among mild AS in our cohort is mid-way between the 1- and 5-year mortality rates in Strange *et al*. This underscores the adverse prognosis for mortality events even in mild AS. Patients with mild AS and moderate AS may die while at this stage of the disease due to other co-morbidities that may not be related to AS itself. In addition, those in moderate AS may progress to severe AS with high risk of death during the follow up period. After adjusting for co-morbidities such as diabetes, ischemic heart disease and traditional prognostic markers of AS such as aortic valve area and transaortic peak velocity, dσ*/dt_*max*_ remained significant associated with poor composite clinical outcomes in moderate AS and severe AS.

This was the first study to look at the novel global LV contractility index, dσ*/dt_*max*_ in pressure overload state. However, it has the inherent limitations of an observational study. Patients with mild AS or moderate AS may have other co-morbidities contributing to mortality beyond the aortic valve disease. We also did not assess patients’ symptoms status at the point of the index echocardiographic study, or if they developed symptoms on subsequent follow-up. In addition, we did not compare dσ*/dt_*max*_ with other more advanced imaging techniques like strain. However, unlike strain, dσ*/dt_*max*_ can be easily derived from conventional echocardiographic parameters without the need for specialized software. Future larger prospective studies can focus on comparing dσ*/dt_*max*_ with advanced imaging such as speckle-tracking analyses.

## Conclusions

Global LV contractility index, dσ*/dt_*max*_ declined with worsening AS severity despite preserved LVEF ≥ 50%. Low dσ*/dt_*max*_ < 2.8 s^−1^ was independently associated with higher composite outcomes of AVR, CCF admissions and all-cause mortality in moderate AS and severe AS.

## References

[1] Freeman RV, Otto CM (2005). Spectrum of calcific aortic valve disease: pathogenesis, disease progression, and treatment strategies. Circulation.

[2] Nkomo VT (2006). Burden of valvular heart diseases: a population-based study. Lancet..

[3] Nishimura RA (2014). American College of Cardiology; American College of Cardiology/American Heart Association; American Heart Association. 2014AHA/ACC guideline for the management of patients with valvular heart disease: a report of the American College of Cardiology/American Heart Association Task Force on Practice Guidelines. J Thorac Cardiovasc Surg.

[4] Lim WY, Ramasamy A, Lloyd G, Bhattacharyya S (2017). Meta-analysis of the impact of intervention versus symptom driven management in asymptomatic severe aortic stenosis. Heart.

[5] Généreux P (2016). Natural history, diagnostic approaches, and therapeutic strategies for patients with asymptomatic severe aortic stenosis. J Am Coll Cardiol.

[6] Tribouilloy C (2015). Lévy F. Low-gradient, low-flow severe aortic stenosis with preserved left ventricular ejection fraction: characteristics, outcome, and implications of surgery. J Am Coll. Cardiology.

[7] Mihaljevic T (2008). Survival after valve replacement for aortic stenosis: implications for decision making. J Thorac Cardiovasc Surg.

[8] Lund O (1997). Left ventricular systolic and diastolic function in aortic stenosis. Prognostic value after valve replacement and underlying mechanisms. Eur Heart J.

[9] Aurigemma GP, Zile MR, Gaasch WH (2006). Contractile behavior of the left ventricle in diastolic heart failure: with emphasis on regional systolic function. Circulation..

[10] Lambert CR, Nichols WW, Pepine CJ (1983). Indices of ventricular contractile state: comparative sensitivity and specificity. Am Heart J.

[11] Legget ME (1999). Usefulness of Parameters of Left Ventricular Wall Stress and Systolic Function in the Evaluation of Patients with Aortic Stenosis. Echocardiography.

[12] Zhong L (2007). Validation of a novel cardiac index of left ventricular contractility in patients. Am J Physiol Heart Circ Physiol.

[13] Jia X (2013). Extent of load-independence of pressure-normalized stress in swine. ExpBiol Med (Maywood)..

[14] Zhong L (2013). Myocardial contractile dysfunction associated with increased 3-month and 1-year mortality in hospitalized patients with heart failure and preserved ejection fraction. Int J Cardiol.

[15] Zhong L, Poh KK, Lee LC, Le TT, Tan RS (2011). Attenuation of stress-based ventricular contractility in patients with heart failure and normal ejection fraction. Ann Acad Med Singapore.

[16] Zhong L (2014). Age and gender- specific changes in left ventricular systolic function in human volunteers. Int J Cardiol.

[17] Lang RM (2015). Recommendations for Cardiac Chamber Quantification by Echocardiography in Adults: An Update from the American Society of Echocardiography and the European Association of Cardiovascular Imaging. J Am Soc Echocardiogr.

[18] Baumgartner H (2017). Recommendations on the echocardiographic assessment of aortic valve stenosis: a focused update from the European Association of Cardiovascular Imaging and the American Society of Echocardiography. Eur Heart J Cardiovasc Imaging.

[19] Nagueh SF (2016). Recommendations for the Evaluation of Left Ventricular Diastolic Function by Echocardiography: An Update from the American Society of Echocardiography and the European Association of Cardiovascular Imaging. J Am Soc Echocardiogr.

[20] Nakano K (1990). Myocardial stiffness derived from end-systolic wall stress and logarithm of reciprocal of wall thickness: contractility index independent of ventricular size. Circulation..

[21] Belcher P, Boerboom LE, Olinger GN (1985). Standardization of end-systolic pressure – volume relation in the dog. Am J Physiol Heart Circ Physiol..

[22] Aurigemma GP, Silver KH, McLaughlin M, Mauser J, Gaasch WH (1994). Impact of chamber geometry and gender on left ventricular systolic function in patients> 60 years of age with aortic stenosis. Am J Cardiol.

[23] Ross J (1985). Afterload mismatch in aortic and mitral valve disease: Implications for surgicaltherapy. J Am Coll Cardiol.

[24] Aurigemma GP, Silver KH, Priest MA, Gaasch WH (1995). Geometric changes allow normal ejection fraction despite depressed myocardial shortening in hypertensive left ventricular hypertrophy. J Am Coll Cardiol.

[25] Rajappan K (2002). Mechanisms of coronary microcirculatory dysfunction in patients with aortic stenosis and angiographically normal coronary arteries. Circulation.

[26] Dahl JS (2011). Noninvasive assessment of filling pressure and left atrial pressure overload in severe aortic valve stenosis: relation to ventricular remodeling and clinical outcome after aortic valve replacement. J Thorac Cardiovasc Surg.

[27] Hein S (2003). Progression from compensated hypertrophy to failure in the pressure-overloaded human heart: structural deterioration and compensatory mechanisms. Circulation..

[28] Duncan AL (2008). Influence of concentric left ventricular remodeling on early mortality after aortic valve replacement. Ann Thorac Surg.

[29] Dahl JS (2012). Global strain in severe aortic stenosis. Circ Cardiovasc Imaging.

[30] Delgado V (2009). Strain analysis in patients with severe aortic stenosis and preserved left ventricular ejection fraction undergoing surgical valve replacement. Eur Heart J.

[31] Kearney LG (2012). Global longitudinal strain is a strong independent predictor of all-cause mortality with aortic stenosis. Eur Heart J Cardiovasc Imaging.

[32] Rydberg E, Gudmunsson P, Kennedy L, Erhardt L, Willenheimer R (2004). Left atrioventricular plane displacement but not left ventricular ejection fraction is influenced by the degree of aortic stenosis. Heart.

[33] Bruch C (2004). Tissue Doppler imaging in patients with moderate to severe aortic valve stenosis: clinical usefulness and diagnostic accuracy. Am. Heart J.

[34] Lafitte S (2009). Impact of impaired myocardial deformations on exercise tolerance and prognosis in patients with asymptomatic aortic stenosis. Eur J Echocardiogr.

[35] Kamperidis V (2014). Left ventricular functional recovery and remodelling in low-flow low-gradient severe aortic stenosis after transcatheter aortic valve implantation. J Am Soc Echocardiogr.

[36] Lafitte S (2009). Impact of impaired myocardial deformations on exercise tolerance and prognosis in patients with asymptomatic aortic stenosis. Eur J Echocardiogr.

[37] Cioffi G (2011). Prognostic effect of inappropriately high left ventricular mass in asymptomatic severe aortic stenosis. Heart.

[38] Lancellotti P (2010). Risk stratification in asymptomatic moderate to severe aortic stenosis: the importance of the valvular, arterial and ventricular interplay. Heart..

[39] Clavel MA (2014). Impact of aortic valve calcification, as measured by MDCT, on survival in patients with aortic stenosis: results of an international registry study. J Am Coll Cardiol.

[40] Dweck MR (2011). Midwall fibrosis is an independent predictor of mortality in patients with aortic stenosis. J Am Coll Cardiol.

[41] Leon MB (2016). PARTNER 2 Investigators. Transcatheter or Surgical Aortic-Valve Replacement in Intermediate-Risk Patients. N Engl J Med..

[42] Spaccarotella C, Mongiardo A, De Rosa S, Indolfi C (2017). Transcatheter aortic valve implantation in patients at intermediate surgical risk. Int J Cardiol..

[43] Strange G (2019). National Echocardiography Database of Australia contributing sites. Poor Long-Term Survival in Patients With Moderate Aortic Stenosis. J Am Coll Cardiol..

